# Impact of mAb-induced A475V substitution on viral fitness and antibody neutralization of SARS-CoV-2 omicron variants in the presence of monoclonal antibodies and human convalescent sera

**DOI:** 10.3389/fimmu.2023.1219546

**Published:** 2023-08-01

**Authors:** Ratchanont Viriyakitkosol, Asawin Wanitchang, Kanjana Srisutthisamphan, Janya Saenboonreung, Chatikorn Boonkrai, Trairak Pisitkun, Anan Jongkaewwattana

**Affiliations:** ^1^ Virology and Cell Technology Research Team, National Center for Genetic Engineering and Biotechnology (BIOTEC), National Science and Technology Development Agency (NSTDA), Pathumthani, Thailand; ^2^ Center of Excellence in Systems Biology, Faculty of Medicine, Chulalongkorn University, Bangkok, Thailand

**Keywords:** SARS-CoV-2, monoclonal antibodies, antibody resistance, A475V, viral fitness

## Abstract

The emergence and rapid evolution of SARS-CoV-2 variants have posed a major challenge to the global efforts to control the COVID -19 pandemic. In this study, we investigated the potential of two SARS-CoV-2 variants, BA.2 and BA.5, to evade neutralization by a human monoclonal antibody targeting the virus’s spike RBD (mAb 1D1). By subjecting the viruses to serial propagation in the presence of the antibody, we found that BA.2 exhibited poor growth, whereas BA.5 regained robust growth with significantly higher kinetics than the parental virus. Genetic analysis identified a single mutation, A475V, in the spike protein of BA.5 that substantially reduced the neutralizing activities of monoclonal antibodies and convalescent sera. In addition, the A475V mutation alone in BA.2 moderately reduced the neutralizing activity but completely abolished the neutralizing effect of mAb 1D1 when F486V or L452R were also present. Our results shed light on the possible evolutionary development of SARS-CoV-2 variants under selection pressure by monoclonal antibodies and have implications for the development of effective antibody therapies and vaccines against the virus.

## Introduction

The outbreak of COVID-19, caused by the SARS-CoV-2 virus, has had a profound impact on global health and the economy. According to the World Health Organization, as of April 2023, the virus had infected more than 760 million people and resulted in more than 6.9 million deaths worldwide. Although the basic biology of SARS-CoV-2 is well established, much remains to be learned about its evolution (1). Like many RNA viruses, SARS-CoV-2 exists as a quasi-species, meaning that a diverse population of variants is constantly present (2). This diversity arises through the mutation of viral genomes, which is driven by stochastic processes. While the majority of mutations are either neutral or deleterious, a small proportion can enhance the fitness of the virus, leading to increased replication rates and pathogenicity. Several factors could influence the evolution of SARS-CoV-2, including the host’s genetic background and immunological status (3, 4), the target tissues or cells (5, 6), and the level of immunity of the population through vaccination or natural infection (3, 7).

Following the emergence of the Omicron variant of SARS-CoV-2, a number of variants containing mutations in the spike protein have emerged, posing a threat to the vaccine-induced immunity that formerly provided robust protection against viral infection. These variants have demonstrated either increased transmissibility or immunological escape and pose a significant threat to public health (8, 9). Omicron (B.1.1.529) and its subvariants (BA.2, BA.4, BA.5) have rapidly become dominant in areas with high population immunity through vaccination, infection, or both. Although the precise mechanism behind the evolution of the antigenically divergent Omicron variant remains unclear, there is a hypothesis that suggests the virus may have adapted to immunocompromised hosts, where it can replicate repeatedly and accumulate mutations that confer a fitness advantage (10, 11). Accumulating evidence also suggests that antibody-mediated selective pressure may have played a role in the emergence of these variants (12, 13).

Several mutations on the spike protein of SARS-CoV-2 Omicron variants have been associated with the virus’s ability to subvert serum neutralization and evade monoclonal antibodies. Recent advances in techniques such as deep mutational high-throughput scanning have allowed for the prediction of important mutations that circumvent the immune system (14). These predictions include mutations such as R346T, L452R, K444T/R, N460Y/K, and F486S/P, which may alter the structure of the spike protein and make it more difficult for antibodies to bind and neutralize the virus. Interestingly, while many of these mutations have been observed in circulating variants, some predicted mutations that could lead to immune-evading mutations are notably absent in the majority of variants to date. Ongoing surveillance and monitoring of SARS-CoV-2 variants is critical to identify new mutations and track their spread, as there is no guarantee that these predicted mutations will not occur in future variants. The identification of these mutations emphasizes the importance of continued research and development of effective vaccines and treatments to combat the ongoing pandemic (15).

In this study, we aimed to examine the evolutionary response of SARS-CoV-2 Omicron variants (BA.2 and BA.5) to monoclonal antibody selection pressure. Our results showed that although monoclonal antibodies targeting the spike RBD were effective in neutralizing both variants, only BA.5 was able to adapt and regain high growth under the influence of suboptimal antibody concentration. Surprisingly, BA.5 evolved with a single A475V substitution. Further investigation indicated that the presence of L452R and F486V on BA.5 was necessary for the spike to function effectively with the A475V mutation. These findings not only enhance our understanding of how SARS-CoV-2 variants evolve under the influence of antibodies, but also provide insights into the general fitness of viruses circulating in nature.

## Materials and methods

### Cell culture and antibodies

Human embryonic kidney (HEK) 293T and HEK-293T cells expressing human ACE2 were cultured in Opti-MEM (Thermo Scientific, Waltham, MA) supplemented with 10% fetal bovine serum (FBS) and 1% penicillin and streptomycin (P/S). Human lung cancer cells (A549) expressing human ACE2 and TMPRSS2 (A549-ACE2-TM2) were obtained from Invivogen (San Diego, CA, USA) and maintained in high-glucose (4,500 mg/ml) Dulbecco’s modified Eagle’s medium (DMEM), supplemented with 10% FBS and 1% P/S, according to the manufacturer’s instructions. The cells were incubated at 37°C in a 5% CO_2_ humidified incubator.

To generate fully human monoclonal antibodies against SARS-CoV-2, human peripheral blood mononuclear cells (PBMCs) were isolated from COVID -19 convalescent patients as previously described (16). Using human hybridoma technology, human hybridoma cells were generated, cultured, and screened for clones producing anti-RBD (wild-type) SARS-CoV-2 antibodies. Subsequently, several human hybridoma clones producing anti-RBD SARS-CoV-2 antibodies were successfully isolated. The panel of SARS-CoV-2 RBD monoclonal antibodies (1D1, 3A6, 1F4) used in this study was derived from these human hybridoma clones (16). Additionally, SARS-CoV-2-specific mouse nucleocapsid monoclonal antibodies were utilized in this study were produced in-house at the BIOTEC monoclonal antibody research team facilities in Thailand.

### Viruses

SARS-CoV-2 variants (BA.2, and BA.5) were obtained from saliva samples of COVID-19 patients in Thailand and propagated using A549-ACE2-TM2 cells as previously described (17). The full-length viral spike gene was then amplified using RT-PCR (Clontech, Mountain View, USA), and the sequences were confirmed through Sanger sequencing. Prior to use in infection experiments, cell culture supernatants were processed to remove cell debris via centrifugation. Titration of viral stocks was performed on A549-ACE2-TM2 cells that were cultured in Opti-MEM supplemented with recombinant trypsin (10 µl/ml) in TrypLE™ Select Enzyme (10X) (Thermo Scientific).

### Expression plasmids

The full-length gene encoding the spike protein of SARS-CoV-2 variants (BA.2, and BA.5) was codon-optimized for high production in mammalian cells. The gene was synthesized and subsequently cloned into the cloning vector pUC57 (Genscript, Piscataway NJ, USA). To enable efficient cloning into the pCAGGS expression plasmid, which was modified to contain both MluI and NotI restriction sites, the synthetic gene was constructed to be flanked by these sites. The In-Fusion cloning kit and a ligation-independent technique (Takara Bio, Mountain View, CA, USA) were used to generate chimeric spikes. Site-directed mutagenesis was also performed on the plasmid via in-fusion cloning as previously described (18). To ensure the efficiency of transfection and protein expression, Western blot analysis was performed on HEK-293T cells transfected with each plasmid prior to further analysis.

### mAb-mediated selection of SARS-CoV-2 variants

In this study, mAb 1D1 was selected for incubation with the virus because of its strong neutralizing activity not only against pre-omicron variants but also against some omicron variants including BA.1 and BA.2. Briefly, SARS-CoV-2 strains BA.2 and BA.5 (at 1000 pfu/ml) were incubated with 10 µl of mAb 1D1 (at a concentration of 500 µg/ml) at 37°C for 1 h. The resulting mixture was then applied to A549-ACE2-TM2 cell monolayers and incubated for another hour at 37°C. The cell monolayers were washed with PBS and then incubated in Opti-MEM, which contained recombinant trypsin (10 µl/ml) and mAb 1D1 (at a concentration of 7.5 µg/ml). After the appearance of cytopathic effects, the supernatants were collected, and the process was repeated using another round of antibody incubation and infection of A549-ACE2-TM2 cells.

### Focus‐forming assay for viral titration

The assay was carried out following a previously described protocol with certain modifications (19). Firstly, 100 µl of virus samples, serially diluted (10-fold), were inoculated into A549-ACE2-TM2 monolayers. After 1 h of incubation at 37°C, samples were removed, and cells were washed with PBS before being cultured in serum-free Opti-MEM supplemented with recombinant trypsin. At 18h post-infection, the supernatant was removed, and cells were fixed with 80% cold acetone for 10 minutes. Subsequently, cells were washed with PBS and blocked in a buffer containing 10% bovine serum albumin (BSA) at room temperature for 1 h. Next, cells were incubated with monoclonal antibodies targeting SARS-CoV-2 N protein in blocking buffer for 1 h at room temperature. After washing, cells were incubated with goat anti-mouse IgG conjugated to alkaline phosphatase (Abcam, Cambridge, UK) for 1 h at room temperature. The wells were then washed and stained with 5-bromo-4-chloro-3-indolyl phosphate (BCIP)/nitroblue tetrazolium (NBT) purchased from Merck (Darmstadt, Germany) for 10 min. Finally, the wells were washed, dried, and imaged using the Chemidoc Imaging System (BIORAD, Hercules, CA). The viral infectivity was expressed as foci forming units (FFUs).

### Pseudovirus neutralization assay

The production of lentiviral pseudoviruses carrying CoV spike was described in previous studies (18, 20). Briefly, to generate these pseudoviruses, a combination of plasmids was used, including the lentivirus backbone that expresses a firefly luciferase reporter gene (pCSFLW), an expression plasmid that expresses HIV-1 structural/regulatory proteins (pCMVR8.91), and pCAGGS that expresses spikes of SARS-CoV-2. HEK293T/17 production cells were seeded 24 h prior to transfection with the plasmids at a concentration of 7.5x10^5^ cells/well in 6-well plates, unless otherwise indicated. The transfection was performed using OptiMEM containing 10 µl polyethyleneimine (PEI). The cells were then incubated with 5% CO_2_ at 37°C. After 12 h of transfection, cells were washed and cultured in DMEM containing 10% FBS. Supernatants containing pseudoviruses were collected 72 h post-transfection, and centrifuged at 1500 x g for 10 min at 4°C to remove cell debris. The collected supernatants were then aliquoted and stored at -80°C. To evaluate the neutralizing activity of monoclonal antibodies or serum samples, a twofold serial dilution of the samples was performed in culture medium (high-glucose DMEM without FBS), starting at 1:40. In a 96-well culture plate, the samples were mixed with pseudoviruses carrying the CoV spike of interest at a ratio of 1:1 vol/vol. The pseudovirus used was set at 1x10^5^ RLU per well. The sample-pseudovirus mixture was then incubated at 37°C for 1 h. Cell suspensions of HEK293T-ACE-2 were then added to each well of CulturPlateTM microplates (PerkinElmer, Waltham, MA, USA). The plates were incubated at 37°C for 48 h, and the neutralizing antibodies were assessed by luciferase activity.

### Western blot analysis

HEK-293T cells were transfected with pCAGGS plasmid encoding the SARS-CoV-2 spike protein. Transfected cells were collected and lysed using 200 µl of mammalian cell lysis buffer (Thermo Scientific). The resulting cell lysates were separated by SDS-PAGE using a 10% acrylamide gel and then transferred onto nitrocellulose membranes (BioRad). The membranes were blocked with 5% non-fat milk in TBS-T buffer for 1 h before incubation with primary antibodies, including polyclonal anti-SARS-CoV-2 spike rabbit antibodies (Sino biological, Beijing, China) or mouse monoclonal anti-β-actin antibodies (Genscript). After washing, the membranes were incubated with secondary antibodies, goat anti-mouse or rabbit IgG conjugated to HRP (Abcam), before being visualized using the Chemidoc Imaging System (BIORAD).

### Immunofluorescence assay

A549-ACE2-TM2 cells were grown in six-well plates and infected with SARS-CoV-2 at 0.1 moi. at 24 h after infection, cells were washed with PBS and fixed with 80% cold acetone for 10 min. Subsequently, the cells were washed and blocked with PBS containing 10% FBS and 1% BSA for 1 h. The cells were then incubated for 1 h with mouse anti-SARS-CoV-2 nucleocapsid antibodies in 10% FBS at a dilution of 1:1000. After washing, goat anti-mouse IgG conjugated to alkaline phosphatase (Abcam) was added in 10% FBS at a dilution of 1:5000 and incubated further for 1 h. Cells were washed and incubated with Vector Red Substrate (Vector Laboratories, Newark, CA, USA) according to the manufacturer’s instructions. Cells were then mounted with with DAPI (Vector Laboratories). Samples were analyzed using a fluorescence microscope.

### Statistical analysis

Statistical analyses were performed using GraphPad Prism 9.0 software. A two-group comparison was performed using Student’s t-test at a significance level of p < 0.05, indicating statistically significant differences between groups.

## Results

### BA.5, but not BA.2, could evolve to antagonize the neutralizing activity of monoclonal antibody 1D1 during serial passages

**Figure 1:**
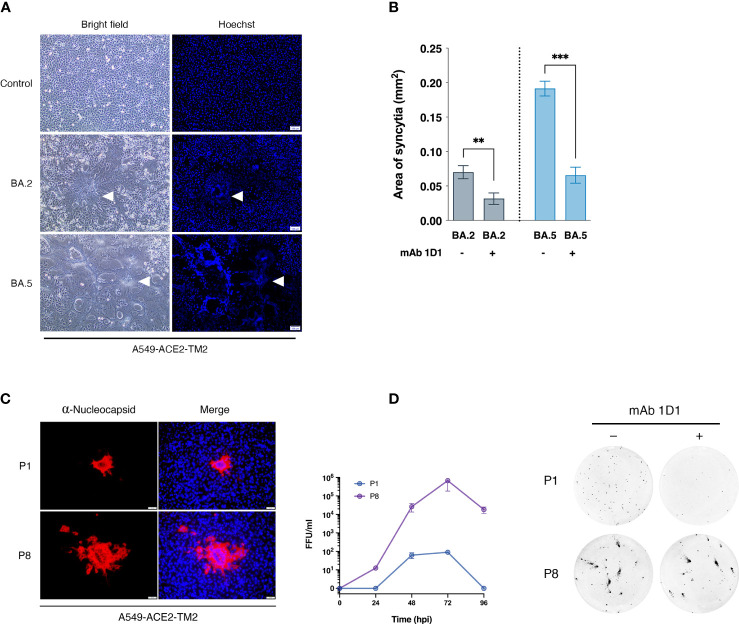
Selection of SARS-CoV-2 variants with monoclonal antibody 1D1. **(A)** Syncytia formation in A549-ACE2-TM2 cells infected with SARS-CoV-2 strains BA.2 and BA.5 in the presence of trypsin. At 24 hpi, syncytia formation was visualized under light microscope, and nuclei were stained with Hoechst and visualized under fluorescence microscope, scale bar 100 µm. Arrows indicate syncytia formation **(B)** Syncytia size of A549-ACE2-TM2 infected with BA.2 and BA.5 in the presence or absence of mAb 1D1 was determined by analyzing syncytia area with ImageJ. Data are presented as mean ± standard deviation (SD) of three independent experiments. **(C)** A549-ACE2-TM2 cells infected with BA.5-P1 and BA.5-P8 for 24 h and analyzed by immunofluorescence staining with antibodies against SARS-CoV-2 nucleocapsid protein. The viral titers of BA.5-P.1 and BA.5-P8 in A549-ACE2-TM2 cells at different time points after infection were examined by focus-forming assay. **(D)** Neutralization of mAb 1D1 against BA.5-P.1 and BA.5-P8 was examined by focus-forming assay. **p < 0.01; ***p < 0.001..

We selected two SARS-CoV-2 strains from saliva samples of COVID -19 patients in Thailand during the Omicron epidemic that occurred from April to July 2022. Sequencing analysis confirmed that these strains possessed spike mutations characteristic of BA.2 and BA.5. After exposure to trypsin, both strains were able to replicate efficiently and form clear syncytia in A549-ACE2-TM2 cells (**Figure 1A**). However, the monoclonal antibody 1D1 (mAb 1D1) was able to effectively neutralize both variants as indicated by significantly reduced size of cell-cell fusion when cultured in the presence of the monoclonal antibody (**Figure 1B**). We then examined whether the individual SARS-CoV-2 strains could confer resistance to the antibody by serially propagating them in the presence of mAb 1D1 (1 µg/ml). While the infectivity of BA.2 decreased and the virus progeny was undetectable by the third passage, BA.5 showed signs of syncytia and regained its high replication by the 8^th^ passage (**Figure 1C**). Comparing the growth kinetics of the parental BA.5 (BA.5-P.1) and that of the later passage (BA.5-P8), we found that the virus of the later passage was capable of replicating more efficiently in A549-ACE2-TM2 cells, with a peak titer at 72 hpi more than 1,000-fold higher than that of the parental virus (**Figure 1C**). To confirm the resistance of BA.5-P8 to mAb 1D1, viruses were incubated with mAb 1D1 and adsorbed onto A549-ACE2-TM2 cells. The number of infected cells detected with the anti-nucleocapsid antibody was significantly higher for BA.5-P8 than for the parental virus BA.5 (**Figure 1D**). These results altogether suggest that selection with monoclonal antibodies not only facilitates the escape of the virus from antibody binding, but also enhances its fitness in host cells.

### A475V is responsible for the mAb 1D1 resistance and enhanced cell-cell fusion

**Figure 2:**
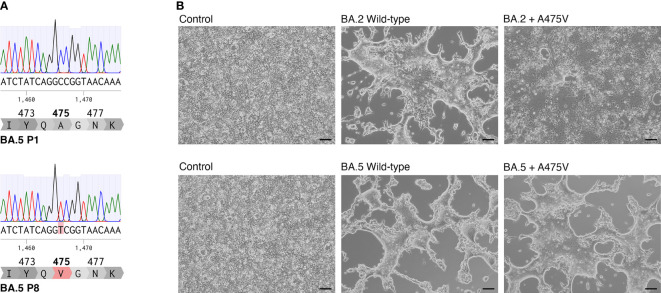
A475V substitution in BA.5 spike protein confers resistance to mAb 1D1 and increases cell-cell fusion. **(A)** Comparison of the full-length spike gene from passages 1 and 8 of BA.5 revealed a single amino acid substitution at position 475, where alanine was replaced by valine (A475V). **(B)** Introduction of A475V substitution into the spike protein of BA.2 and BA.5 resulted in differential effects on cell-cell fusion activity in HEK-293T-ACE2 cells. Representative data from three independent experiments are shown.

To investigate the possible mutation of the BA.5 spike protein responsible for evading neutralization by mAb 1D1, we performed a comparative analysis of the full-length spike gene from passages 1 and 8. Our results, shown in **Figure 2A**, revealed a single amino acid substitution at position 475, where alanine was substituted by valine (A475V). Notably, we further analyzed the virus at passage 15 and observed that the sequence remained unchanged from that of passage 8, suggesting that the A475V substitution alone may be responsible for resistance to neutralization by mAb 1D1. It is, however, important to note that alterations beyond the spike protein that were not examined in this study may also play a role in the observed viral properties, including those unrelated to antibody resistance. To determine the potential effects of the A475V substitution, we introduced the A475V substitution into the spike protein of BA.2 and BA.5 and transfected the constructs into HEK-293T cells overexpressing ACE2 (HEK-293T-ACE2) and analyzed cell-cell fusion activity. Our results, as depicted in **Figure 2B**, demonstrated that the presence of A475V in cells expressing BA.2 spike resulted in reduced cell-cell fusion activity. In contrast, cells expressing wild-type BA.5 spike and that with A475V exhibited comparable levels of cell-cell fusion, suggesting that the effects of A475V substitution were more pronounced in BA.2 in terms of impairing cell-cell fusion. These results suggest that A475V substitution may differentially affect the infectivity of BA.2 and BA.5, possibly by promoting cell-cell fusion or receptor binding affinity.

### Pseudovirus carrying BA.5 spike with A475V could significantly evade neutralizing activity of monoclonal antibodies and convalescent sera

**Figure 3:**
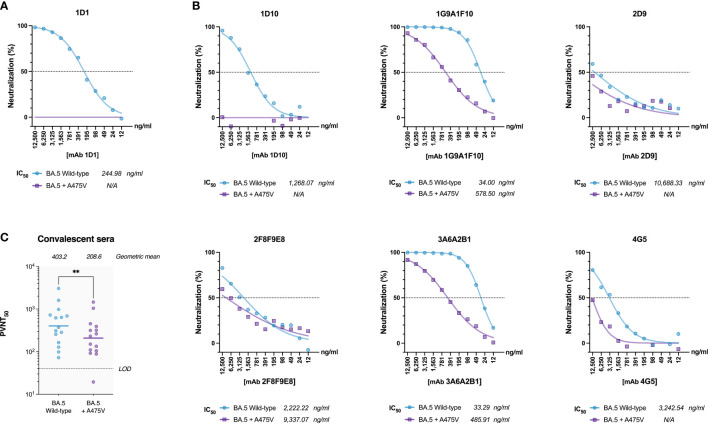
A475V mutation confers resistance to mAb 1D1 and other RBD-specific monoclonal antibodies. **(A)** Neutralization by mAb 1D1 of pseudoviruses carrying either the wild-type BA.5 spike or the one with the A475V mutation. **(B)** Neutralization by other RBD-specific monoclonal antibodies of pseudoviruses carrying either the wild-type BA.5 spike or the one with the A475V mutation. **(C)** Neutralization of pseudoviruses carrying either the wild-type BA.5 spike or the one with the A475V mutation by convalescent sera from vaccinated individuals who experienced breakthrough during the BA.2 and BA.5 waves. Geometric mean neutralization titers are indicated. LOD, Limit of detection. GraphPad Prism 9.0 was used to determine IC_50_ values, analyze statistical significance, and generate all graphs. The error bars are means ± standard error. ns, P ≥ 0.05; **P < 0.01.

To investigate the potential resistance conferred by the A475V mutation against mAb 1D1, we examined the neutralizing activity of mAb 1D1 against pseudoviruses carrying either the wild-type BA.5 spike or the one with the A475V mutation. Our results showed that although mAb 1D1 effectively neutralized pseudoviruses carrying the wild-type BA.5 spike (IC50 = 244.98 ng/ml), it failed to neutralize pseudoviruses carrying the A475V mutation (**Figure 3A**). Remarkably, the presence of A475V affected not only the neutralizing activity of mAb 1D1 but also the neutralizing activity of other monoclonal antibody clones targeting the RBD we tested in this study (**Figure 3B**). In addition, we examined the neutralizing activity of convalescent sera obtained from vaccinated individuals who experienced breakthrough infections during the BA.2 and BA.5 waves against the same panel of pseudoviruses. Our results showed that the presence of A475V on the BA.5 spike resulted in a 1.93-fold decrease in the geometric mean neutralization titer (**Figure 3C**). Collectively, these results suggest that A475V could enable the BA.5 to evade neutralization of spike-specific antibodies.

### BA.2 spike with A475V does not confer complete resistance against mAb 1D1

**Figure 4:**
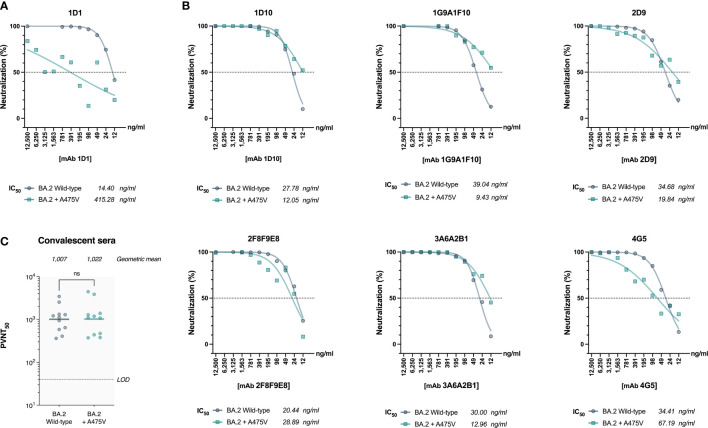
The effect of A475V mutation on the neutralizing activity of mAb 1D1 and convalescent sera against BA.2 spike. **(A)** Neutralizing activity of mAb 1D1 against pseudoviruses carrying either the wild-type BA.2 spike or that with A475V substitution. Shown is the IC50 value of each group. **(B)** Neutralizing activity of RBD-specific monoclonal antibodies against pseudoviruses carrying BA.2 spike and that with A475V substitution. The IC_50_ values of each group are shown. **(C)** Neutralizing activity of convalescent sera against pseudoviruses carrying either the wild-type BA.2 spike or that with A475V substitution. Shown is the geometric mean neutralizing titer (GMT) of each group.

Based on our observation that BA.2 cannot be recovered in serial passages in the presence of mAb 1D1, we speculated that the A475V substitution in BA.2 spike might not be sufficient to enable BA.2 to escape mAb 1D1 neutralizing activity. To test this hypothesis, we generated pseudoviruses carrying the wild-type BA.2 spike and the spike with the A475V mutation and examined their susceptibility to neutralization by mAb 1D1. Our results, shown in **Figure 4A**, indicate that unlike BA.5 spike carrying the A475V mutation, BA.2 spike with the same mutation can still be neutralized by mAb 1D1, albeit with a significant increase in IC50 compared with wild-type BA.2 (415.28 ng/ml versus 14.40 ng/ml). We also observed no significant changes in the neutralizing activity of other RBD-specific monoclonal antibodies against the pseudoviruses carrying the A475V mutation (**Figure 4B**). In addition, we found that the neutralizing activity of convalescent sera against the pseudoviruses carrying the BA.2 spike with A475V was similar to that against the wild type BA.2 (**Figure 4C**). Taken together, our results suggest that the presence of A475V on the BA.2 spike alone is not sufficient to confer resistance to monoclonal antibodies or convalescent sera.

### A475V and BA.5-specific mutations (L452R, F486V and R493Q) are required for BA.2 spike to evade mAb 1D1 neutralization

**Figure 5:**
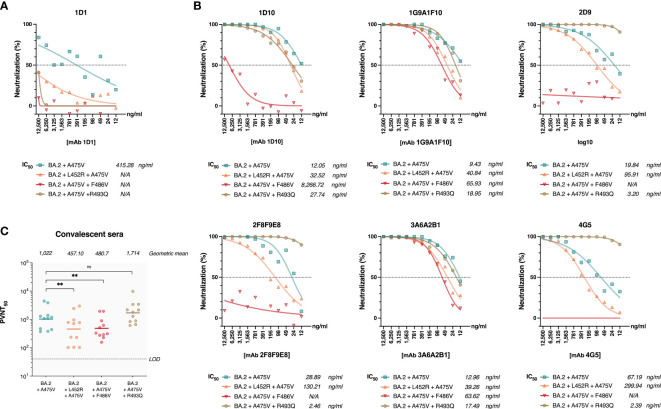
Impact of BA.5-specific RBD mutation on neutralization of pseudoviruses bearing BA.2 spike protein with A475V mutation. **(A)** Neutralizing activity of mAb 1D1 against pseudoviruses carrying BA.2-A475V and introduced L452R, F486V, or R493Q. Shown is the IC50 value of each group. **(B)** Neutralizing activity of RBD-specific monoclonal antibodies against pseudoviruses carrying BA.2-A475V and introduced L452R, F486V, or R493Q. The IC50 values of each group are shown. **(C)** Neutralizing activity of convalescent sera against pseudoviruses carrying BA.2-A475V and introduced L452R, F486V, or R493Q. Shown is the geometric mean neutralizing titer (GMT) of each group. The error bars are means ± standard error. ns, P ≥ 0.05; **P < 0.01.

The receptor-binding domains (RBDs) of the BA.2 and BA.5 spikes differ only by only three amino acids (L452R, F486V, and R493Q). However, we observed markedly differences in the effects of the A475V mutation on these variants. We thus hypothesized that one or more of the amino acids in the RBD of BA.5 might be responsible for the distinct behavior of the A475V mutation in this variant. To this end, we introduced L452R, F486V, or R493Q into the spike protein of BA.2 that carried the A475V mutation. We then examined whether pseudoviruses carrying these constructs could resist neutralization by mAb 1D1 in a manner similar to BA.5-A475V. Our results showed that the presence of any single L452R, F486V, or R493Q in the BA.2-A475V spike enabled pseudoviruses to evade neutralization by mAb 1D1 with F486V being the most effective at escaping the neutralizing activity of mAb 1D1 (**Figure 5A**). Interestingly, when tested against other RBD monoclonal antibodies, BA.2-A475V with F486V was able to escape 4 of 6 monoclonal antibodies, whereas the variant with L452R only moderately increased the IC_50_ against some clones and the variant with R493Q could not evade the antibodies and was even completely neutralized by some clones (**Figure 5B**). Moreover, the presence of L452R and F486V on the BA.2-A475V spike reduced the neutralizing ability of convalescent sera at a comparable level. However, the variant with R493Q did not show any difference in terms of neutralization compared with BA2-A475V alone (**Figure 5C**). Taken together, our results suggest that the combination of BA.5-specific mutations and A475V mutations in the spike protein of the BA.2 variant allows the virus to effectively evade antibody-mediated inhibition and resist neutralization by mAb 1D1 and other SARS-CoV-2-specific antibodies in convalescent sera.

### The presence of L452R and F486V could promote cell-cell fusion activity of BA.2-A475V

**Figure 6:**
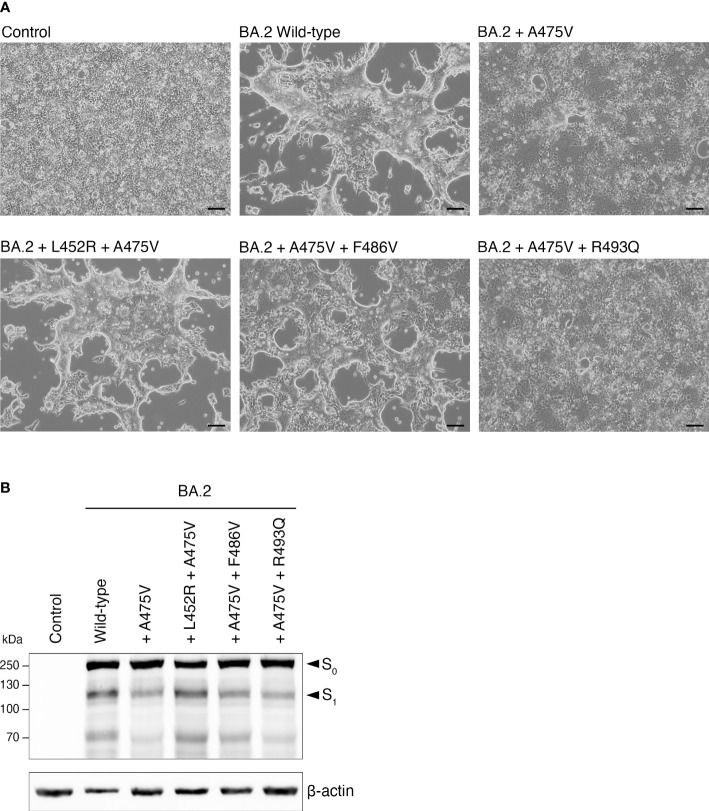
Effects of BA.5-specific mutations on cell-cell fusion activity induced by BA.2-A475V. **(A)** HEK-293T-ACE2 cells were transfected with plasmids expressing BA.2-A475V spike protein with each BA.5-specific mutation in the presence of trypsin. Cell-cell fusion was observed under a light microscope. Representative data from three independent experiments are shown. **(B)** Western blot analysis of protein expression and cleavage pattern of spike proteins using lysates from HEK-293T-ACE2 transfected with plasmids expressing BA.2-A475V and those with BA.5-specific mutations. Rabbit polyclonal antibodies specific for SARS-CoV-2 spike were used to detect S0 and S1, as indicated by arrows. Immunoblots were probed with an anti-beta-actin antibody as a loading control.

We next examined the effects of the BA.5-specific mutations on BA.2-A475V with respect to cell-cell fusion activity. To this end, we performed transfection with plasmids expressing each BA.2 spike protein variant in HEK-293T-ACE2 cells. Our results showed that the presence of either the L452R or the F486V mutation in BA.2-A475V substantially increased cell-cell fusion activity, similar to BA.5-A475V-transfected cells (**Figure 6A**). However, the R493Q mutation in BA.2-A475V did not significantly affect cell fusion activity (**Figure 6A**). We also examined the protein expression and cleavage patterns of BA.2-A475V with each BA.5-specific mutation and found that protein expression was comparable in all constructs, whereas the constructs with L452R and possibly F486V exhibited higher expression of S1. These mutations may therefore render BA.2-A475V more susceptible to proteolytic cleavage (**Figure 6B**). Our results suggest that the combined influence of the BA.5-specific mutations (L452R and F486V) and the A475V mutations may increase the binding affinity between the BA.2 spike protein and the ACE2 receptor, resulting in spike cleavage and increased cell-cell fusion activity.

### Bioinformatics-based prediction suggests that A475V could improve immune escape of BA.5

**Figure 7:**
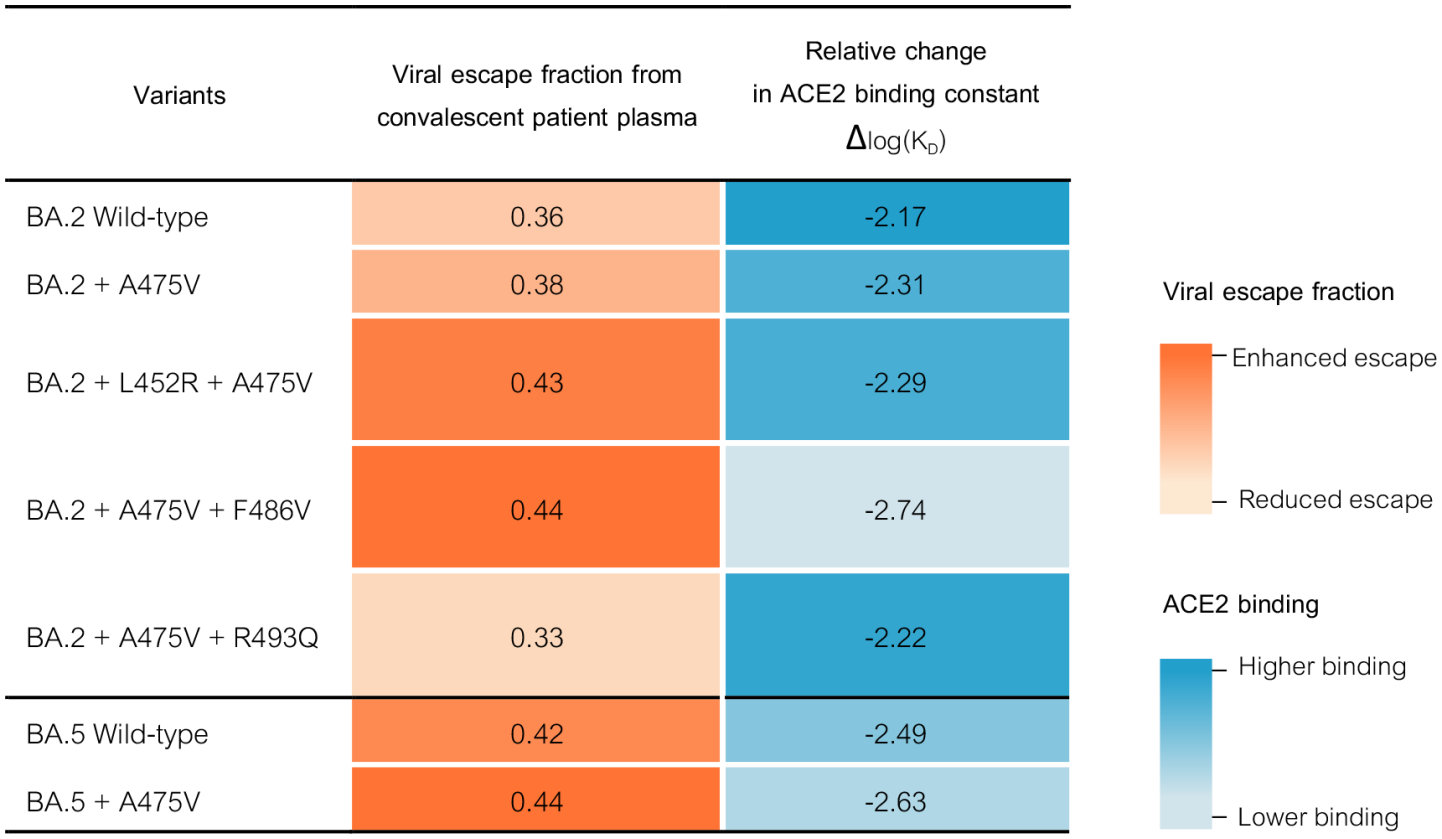
SpikePro, a structure-based computational program, was used to predict the effect of A475V substitution on the fitness of the BA.2 and BA.5 variants. In addition, the effect of the BA.5-specific mutations on BA.2 immune escape and ACE2 affinity binding were predicted.

To investigate the impact of A475V on the overall fitness of BA.5, we utilized SpikePro, a recently developed structure-based computational tool designed to predict the viral fitness of a variant based on its spike protein sequence (21, 22). Our analysis of the prediction data revealed that the presence of a single A475V substitution in the spike protein resulted in improved immune escape from convalescent plasma (**Figure 7**). In addition, the high-throughput mutagenesis data (23) indicated that the A475V substitution could lead to a change in the spike protein ACE2 binding constant (Δlog(KD)) from -2.49 to -2.63, suggesting a higher affinity for ACE2 binding. Interestingly, the predicted ACE2-binding affinity of BA.2-L452R-A475V was slightly lower than that of BA.2-A475V. This is a marked difference from BA.2-A475V-F486V, which had a higher ACE2 binding affinity. However, both BA.2-L452R-A475V and BA.2-A475V-F486V exhibited a higher viral escape fraction from convalescent plasma, with a value comparable to that of BA.5-A475V. Overall, the bioinformatics tool results were consistent with our findings, suggesting that the inability of BA.2 to evolve and harbor the A475V mutation under mAb 1D1 selection was due to its suboptimal fitness.

## Discussion

SARS-CoV-2 is a highly infectious virus that evolves rapidly, exhibiting multiple mutations particularly in the spike protein that contribute to its adaptability and increased transmissibility. The pace of evolution raises concerns that the virus could evolve to circumvent immunity generated by vaccination or natural infection (24, 25). Evidence is accumulating that SARS-CoV-2 can undergo substantial changes in immunocompromised patients, in whom antibody responses are often limited (10, 11). This suggests that the virus is under pressure to evolve in response to suboptimal antibodies, which may lead to the emergence of new variants. For example, several studies have shown that the virus has undergone extensive evolution in immunocompromised individuals with persistent SARS-CoV-2 infection, with multiple mutations in the spike protein that can alter its antigenic properties (12, 26–28). Moreover, these mutations differed from those observed in the general population, suggesting that the virus evolves in response to the immune environment of these patients. In addition, prolonged viral replication in immunocompromised individuals may also result in new variants with altered virulence or transmissibility (29).

This study aims to investigate whether two different SARS-CoV-2 variants from the Omicron lineage (BA.2 and BA.5) can develop resistance to neutralizing antibodies, particularly mAb 1D1, when propagated under suboptimal conditions. Although mAb 1D1 effectively neutralized both variants, BA.2 did not thrive in A549-ACE2-TM2 cells, whereas BA.5 quickly regained high growth rates after only a few passages in the presence of the antibody. Subsequent passages showed that BA.5 replicated more efficiently in host cells, suggesting that it became more fit during adaptation. Further sequence analysis revealed that a single amino acid substitution (A475V) on the BA.5 spike likely enables the virus to evade the neutralizing effects of mAb 1D1 and convalescent sera. In contrast, A475V alone does not confer BA.2 resistance to mAb 1D1 or evade the neutralizing effects of convalescent sera. As expected, we found that double mutations of A475V with BA.5-specific mutations (L452R, F486V, or R493Q) in BA.2 were required to confer antibody resistance similar to that observed with BA.5-A475V. This may explain why BA.2 cannot survive serial passages in the presence of mAb 1D1, which requires at least two concurrent mutations at the key spike residue to escape. Of note, we attempted to propagate various pre-Omicron variants including D614G, Alpha, Beta and Delta along with Omicron BA.1 in the presence of mAb 1D1. Unfortunately, we were unable to find an optimal concentration of mAb 1D1 that would allow viruses to escape its effects. Even at nanomolar concentrations, all tested viruses were unable to tolerate the presence of mAb 1D1 and resumed their growth in culture.

It is worth noting that in the early phase of the pandemic, when the Wuhan-like SARS-CoV-2 virus was prevalent, the A475V mutation, along with D614G and E484Q, was identified as a crucial mutation that decreased the neutralizing activity of convalescent plasma samples (30). Some studies also showed that the highly potent monoclonal antibody B38 had 10,000-fold reduction in neutralizing activity against pseudoviruses with the A475V mutation compared to the parental virus’s spike (31). Consequently, A475V was included in deep mutation scan analyzes as one of the mutations that could confer resistance to neutralizing antibodies (32). Nevertheless, the major circulating SARS-CoV-2 variants, including those derived from BA.2 and BA.5, lack A475V as a critical mutation. Based on GISAID database, some SARS-CoV-2 isolates in both the BA.2 and BA.5 variants do carry A475V. However, it remains uncertain whether these mutations arise in immunocompromised individuals or in individuals undergoing therapeutic antibody treatment, as they are not frequently observed across majority of the viral genetic sequences. This suggests that the emergence of new variants with mutations that allow the virus to evade neutralizing antibody responses may not be due solely to pressure from pre-existing antibodies in the host. It is also possible that A475V gives the virus a selection advantage in the laboratory, where it is under antibody pressure. However, this advantage may not apply to the real world, where other factors such as host immune responses, competition with other viral strains, and transmission dynamics could play an important role. To better understand the prevalence or absence of the A475V mutation in natural populations, further research may be needed, such as *in vivo* experiments and virus sequence analysis from natural populations.

Recently, X-ray crystallography was used to identify the epitope of mAb 1D1, and it was shown that A475 of SARS-CoV-2 RBD forms a hydrogen bond with the asparagine residue (N) 32 of the 1D1 Fab (16). Therefore, it is likely that substituting alanine with valine at this position disrupts the interaction, reducing the ability of mAb 1D1 to bind to the BA.5 spike and prevent interaction with the ACE2 receptor. Interestingly, our findings reveal that mAb 1D1 retains its neutralizing ability towards the BA.2 spike even when hydrogen bonding between A475 and N32 is disrupted by the A475V mutation. Conversely, the presence of BA.5-specific mutations, particularly L452R and F486V, in combination with A475V substantially diminishes the neutralizing activity of mAb 1D1, suggesting that these mutations may cause further disruption of the interaction between mAb 1D1 and RBD via other key epitope residues. Further investigation by molecular docking or x-ray crystallography is required to determine the mechanism by which L452R or F486V and A475V interact in the BA.2 spike to prevent the neutralizing activity mediated by mAb 1D1. Importantly, our results demonstrate that the presence of A475V impairs not only the neutralizing activity of mAb 1D1 but also other monoclonal and convalescent sera that target RBD. This implies that A475V could impact antibody binding via an interaction different from that of mAb 1D1.

In conclusion, our study demonstrates that the A475V mutation in the BA.5 spike likely arose due to selective pressure from mAb 1D1, providing resistance to both mAb 1D1 and convalescent sera. However, the infrequent occurrence of A475V in other highly transmissible variants suggests that additional factors may be necessary for optimal growth. Our findings thus underscore the importance of closely monitoring viral mutations, particularly when monoclonal antibodies are employed as therapy. The emergence of new mutations that enable the virus to evade immune responses could have severe public health implications. By identifying and tracking these mutations, we can better understand the evolution of the virus and take steps to develop more effective treatments and prevention strategies.

## Data availability statement

The raw data supporting the conclusions of this article will be made available by the authors, without undue reservation.

## Author contributions

RV, and AJ designed the experiment protocol. CB and TP produced and characterized monoclonal antibodies. RV, AW, KS and JS performed experiments and data analysis. RV and AJ wrote the manuscript, created the figures and tables. AJ supervised the research and finalized the manuscript. All authors contributed to the article and approved the submitted version.
